# A Review of Video-Based Monitoring Systems for Geohazard Early Warning

**DOI:** 10.3390/s25237385

**Published:** 2025-12-04

**Authors:** Haoran Dong, Shuzhong Sheng, Chong Xu

**Affiliations:** 1 School of Geophysics and Space Exploration, East China University of Technology, Nanchang 330013, China; 2024120103@ecut.edu.cn; 2 National Institute of Natural Hazards, Ministry of Emergency Management of China, Beijing 100085, China; 3 Key Laboratory of Compound and Chained Natural Hazards Dynamics, Ministry of Emergency Management of China, Beijing 100085, China

**Keywords:** video-based monitoring system, geohazards, monitoring and early warning

## Abstract

In recent years, video-based monitoring systems have been widely adopted across multiple domains and have become particularly vital in geohazard monitoring and early warning. These systems overcome the inherent limitations of conventional monitoring techniques by enabling real-time, non-contact, and intuitive visual observation of geologically hazardous sites. With the integration of machine learning and other advanced analytical methods, video-based systems can process and interpret image data in real time, thereby supporting rapid detection and timely early warning of potential geohazards. This substantially improves both the efficiency and accuracy of monitoring efforts. Drawing on domestic and international research, this article provides a comprehensive review of video-based monitoring technologies, machine learning–driven video image processing, and multi-source data fusion approaches. It systematically summarizes their underlying technical principles and applications in geohazard monitoring and early warning, and offers an in-depth analysis of their practical advantages and future development trends. This review aims to serve as a valuable reference for advancing research and innovation in this field.

## 1. Introduction

Geohazards, a major category of natural disasters, are globally pervasive and pose substantial risks to human society and the natural environment. In the context of accelerating global climate change and increasingly intensive human disturbances to natural systems, both the frequency and severity of geohazards have shown a rising trend [1,2]. Consequently, accurate and timely monitoring and early warning are essential for mitigating disaster impacts and safeguarding public safety.

Conventional geohazard monitoring methodologies—typically characterized by point-based observations and physical parameter measurements—primarily include two categories: instrument-based techniques (e.g., crack meters, total stations, piezometers, and rain gauges) and manual field inspections. However, these approaches suffer from limitations in spatial coverage, temporal resolution, and data accuracy, making them insufficient for complex and rapidly evolving geohazard environments [3,4]. Therefore, developing an efficient and reliable monitoring and early warning system has become a pressing need for safeguarding public safety and reducing disaster-related losses [5].

In the field of geohazard mitigation, video-based monitoring systems are increasingly becoming an indispensable tool due to their unique advantages. Unlike previous reviews that treat video imaging as a standalone sensing technique, this study positions video monitoring within the broader ecosystem of multi-source geohazard observation, emphasizing its synergistic role with remote sensing, IoT sensors, and numerical models. A distinctive contribution of this review lies in its systematic classification of video-driven monitoring architectures from both an artificial-intelligence perspective and a computing-architecture perspective—an angle that is largely absent in earlier literature. By highlighting how machine learning and multimodal data fusion unlock the latent value embedded in video streams, this review demonstrates the ongoing paradigm shift in the field: from traditional “geographic-information-driven” approaches toward a new generation of “visual-intelligence-driven” geohazard monitoring and early-warning systems.

In contrast to conventional monitoring methods, video-based systems allow real-time, visual observation of dynamic processes at geohazard sites [6]. Whether capturing the sliding processes of landslides or tracking the flow direction and velocity of debris flows, these systems provide clear visualizations for monitoring personnel, thereby offering critical support for rapid assessment of disaster severity and facilitating emergency decision-making [7,8].

At the data application level, video-based monitoring systems can be integrated with instruments such as GNSS receivers, tiltmeters, rain gauges, and InSAR to generate more comprehensive and accurate datasets for geohazard analysis [9]. When combined with artificial intelligence (AI) and computer vision technologies, these systems enable automated identification of geohazard features, substantially improving monitoring efficiency and accuracy [10,11]. For instance, when rainfall reaches a critical threshold, video data can be used to verify whether surface displacement has occurred, thereby increasing the reliability of landslide early warning. The integration and intelligent analysis of such multi-source data allow monitoring systems to capture the evolution of geohazards more comprehensively and precisely, providing stronger data support for early warning and disaster mitigation.

Video-based monitoring systems exhibit strong adaptability in complex and dynamic environments. By integrating multiple platforms—including fixed cameras, UAVs, and mobile patrol vehicles—these systems achieve flexible spatial coverage across diverse terrains. They support both continuous 24/7 surveillance of critical zones and rapid deployment for emergent monitoring needs, thereby meeting the diverse requirements of geohazard management [12]. During extreme weather events, monitoring personnel can utilize video surveillance systems to observe field conditions in real-time, thereby significantly reducing sampling intervals [13]. In topographically complex mountainous regions, UAV-mounted video sensors can capture multi-angle, high-resolution imagery, providing valuable data for 3D geological hazard modeling and analysis. Moreover, advancements in video technology further enhance the quality and processing efficiency of aerial imagery, thereby strengthening capabilities for geohazard monitoring and early warning [14].

In terms of system integration, video-based monitoring platforms leverage their technical strengths to support long-distance surveillance. Through real-time acquisition and transmission of high-definition video data, they enable precise geohazard monitoring and analysis [15,16,17]. The platforms also incorporate functionalities such as data sharing, alarm interoperability, and remote pan–tilt–zoom control, collectively forming a complete closed-loop process from monitoring to early warning. This integrated technical framework provides strong support for geohazard mitigation, significantly enhancing the efficiency, accuracy, and reliability of monitoring and early warning operations.

This article provides a systematic review of the current applications and recent advancements of video-based monitoring systems in geohazard monitoring and early warning. By synthesizing domestic and international research, it examines the technical advantages and future development prospects of these systems in monitoring geohazards such as landslides and debris flows. The review aims to offer a valuable technical reference for geohazard monitoring and early warning, while promoting technological integration and innovation within the field.

This article retrieves Chinese and English literature from Google Scholar, the Web of Science, and CNKI databases over the past decade, using key terms such as video, image, geological disasters, monitoring, and early warning, along with their combinations and extensions. The majority of the retrieved documents are journal articles, with a few being theses. Meanwhile, in the process of collecting documents on the application of video image monitoring technology in geological disaster monitoring and early warning, most of the documents retrieved by the above retrieval methods are related to China, and a few are from other regions.

## 2. Composition and Technology of Video-Based Monitoring System

### 2.1. Composition of Video-Based Monitoring System

**Figure 1:**
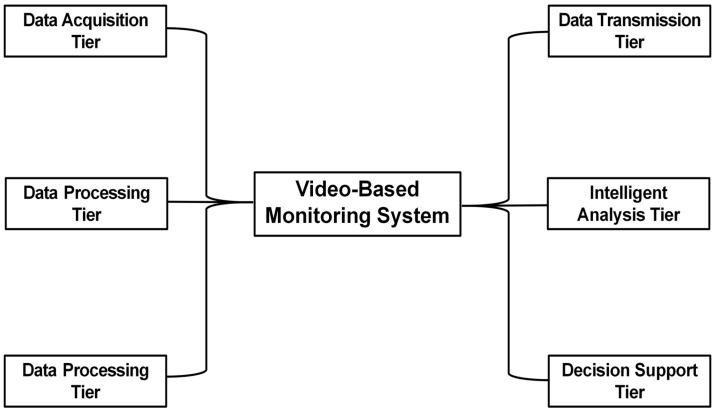
Hierarchical video monitoring system architecture.

In the context of geohazard monitoring and early warning, video-based monitoring systems adopt a multi-tiered, modular architecture [18], integrating functionalities such as data acquisition, transmission, processing, analysis, and early warning. As illustrated in Figure 1, the system can be divided into six modules: data acquisition layer, data transmission layer, data processing layer, intelligent analysis layer, early warning release layer, and decision support layer.

Data Acquisition Tier: Video acquisition devices (e.g., HD cameras, infrared cameras, panoramic cameras) are deployed in geohazard-prone areas or mounted on UAVs and satellites to monitor surface deformations. These devices capture real-time data and transmit it to the back end via the data transmission layer.

Data Transmission Tier: This layer receives data from the acquisition layer and transmits it to the data processing layer using Ethernet, 4G/5G, Wi-Fi or other communication methods.

Data Processing Tier: Incoming data is preprocessed using techniques such as image enhancement, denoising, and segmentation. The processed data is then provided to the intelligent analysis layer according to its requirements.

Intelligent Analysis Tier: This layer actively retrieves data from the processing layer and uses deep learning models to automatically extract disaster features, accurately track targets in complex scenes, and process time-series data to capture spatiotemporal characteristics of hazards. Machine learning models, such as random forests or support vector machines, are then employed to predict the occurrence and development of hazards. The analysis results are sent to both the early warning and decision support layers.

Early Warning Tier: This layer receives event information from the intelligent analysis layer. When disaster indicators exceed predefined thresholds, the system immediately issues early warning alerts.

Decision Support Tier: Analysis results from the intelligent analysis layer are aggregated and compared with historical data. The integrated information is presented on a visual interface to support scientific and informed emergency response planning.

### 2.2. Video-Based Monitoring Technology

Video-based monitoring technology refers to the use of cameras and related devices to acquire image or video data, which is subsequently processed, analyzed, and interpreted through computer-based methods. In the context of geohazard monitoring and early warning, such systems leverage their advantages in non-contact measurement and real-time visualization of field conditions, substantially enhancing the efficiency and accuracy of early warning efforts. This section categorizes video-based monitoring technologies into three major types—close-range photogrammetry, real-time monitoring, and UAV-based remote sensing—and synthesizes recent methodological advancements and applications in geohazard monitoring and early warning.

#### 2.2.1. Close-Range Photogrammetry

Close-range photogrammetry is a representative non-contact monitoring technique that involves capturing images of target objects within a specified distance using photographic equipment (e.g., high-definition cameras, digital cameras), followed by image parsing and intuitive characterization of the object’s surface features [19]. Compared with conventional methods, this technology offers distinct advantages including non-invasiveness and high information density [20,21].

Landslides, characterized by sudden onset and severe impact, require precise monitoring of deformation and other critical features for effective disaster prevention and mitigation. Wu [22] implemented a multi-baseline digital close-range photogrammetric system to monitor slope deformation, demonstrating its feasibility and effectiveness for high-precision monitoring and providing a novel technical solution for geohazard surveillance. Wang [23] applied photogrammetric techniques to high-risk slopes in the Three Gorges Reservoir area, acquiring sequential images to construct a three-dimensional spatial model, thereby enabling visual monitoring of unstable slopes. Qing [24] established a wireless connection between cameras monitoring high-risk slopes and a measurement and control center, facilitating real-time reception, storage, and processing of image data. Additionally, a 3D simulation model of slope landslides was developed for high-precision numerical simulations. Across five application sites, measured minimum errors were 0.0004 mm, maximum errors consistently remained below 2 mm, the maximum root mean square error was 0.6108 mm, and the mean error stayed under 1.2 mm, illustrating that smaller errors correspond to higher early-warning accuracy. These studies highlight the substantial potential of close-range photogrammetry in landslide monitoring, particularly for high-precision deformation measurement, visual modeling, and stability assessment, thereby providing robust technical support for accurate early warning.

Rockfall hazards, characterized by their sudden onset, high velocity, and devastating impact, require precise monitoring of their kinematic processes, which is critical for effective disaster prevention and mitigation. In the context of rock mass structure and collapse monitoring, Wang [25] utilized a digital panoramic borehole camera system to acquire high-resolution borehole images. Image preprocessing techniques in both RGB and HSV color spaces were applied to mitigate the impact of illumination variance on image quality. Furthermore, digital image unwrapping technology was employed to analyze structural characteristics of the borehole wall in detail, providing technical support for investigating changes in rock mass structure. Zhang [26] documented a rock toppling event on 20 June 2023, in Guizhou, China, using fixed on-site cameras. By integrating UAV surveys, particle size distribution analysis, and numerical simulations, the study revealed the kinematic processes and underlying mechanisms of the rockfall, providing a scientific basis for hazard prediction and risk assessment under similar geological conditions. Collectively, these studies demonstrate that close-range photogrammetry, combined with multiple analytical approaches, effectively characterizes rock mass structural changes and rockfall kinematics, supporting hazard prediction and risk assessment.

**Figure 2:**
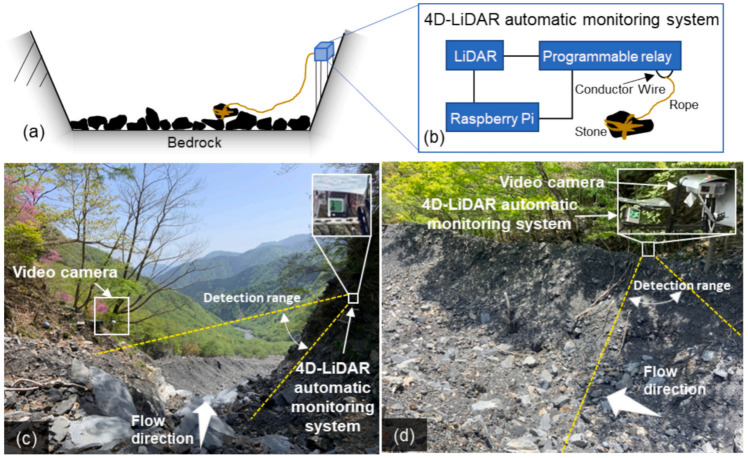
Debris flow monitoring: extracting debris flow features with a 4D-LiDAR video camera [27]. (**a**) Schematic layout of the 4D-LiDAR monitoring system; (**b**) Component framework of the 4D-LiDAR monitoring system; (**c**) Upstream scene of the deployed 4D-LiDAR system; (**d**) Downstream scene of the video-based collaborative monitoring system.

Debris flows, characterized by their sudden onset, devastating impact, and extensive affected areas, require monitoring of their movement pathways and depositional processes as a critical component of disaster prevention and mitigation strategies. To capture the dynamic characteristics of the whole process of debris flow from initiation and migration to accumulation, Kaneko Tatsuki [27] designed a coordinated observation system. Based on the different behaviors of debris flows in the transport and deposition zones, 4D-LiDAR automatic observation systems and video cameras were, respectively, deployed upstream and downstream of the Ohya landslide area to construct a complete disaster process evolution map (Figure 2).

#### 2.2.2. Real-Time Monitoring

**Figure 3:**
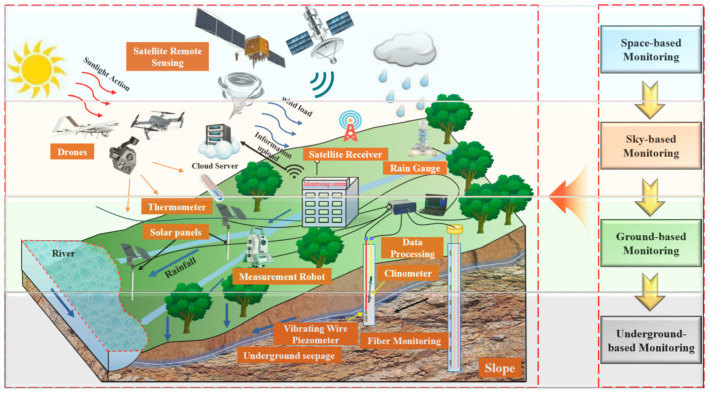
Multi-dimensional landslide monitoring system: integrating space, sky, ground and underground technologies to achieve comprehensive real-time monitoring [30].

Real-time monitoring technology enables automated, long-term surveillance and early warning of hazard-prone areas through the deployment of video acquisition devices integrated with advanced image processing and data analysis techniques, thereby providing timely and accurate information support for disaster prevention and mitigation. Zhao [28] deployed simple video acquisition devices in the hazard-prone area to obtain real-time field imagery. By integrating AI-based early warning algorithms, this system achieves automated long-term monitoring and early warning for the hazard zone. Nie [29], through real-time video monitoring and analysis of video image data, utilized mathematical morphology methods (e.g., dilation, erosion, opening, and closing) and edge detection algorithms (e.g., the Canny operator) to extract the scarp crack curve of the landslide. This approach enabled the three-dimensional display of the scarp crack curve of the landslide, more intuitively reflecting the movement trajectory of the crack curve. In response to the intricate internal architecture and diverse triggering mechanisms of the Xinpu landslide in the Three Gorges area, Cheng [30] established a multi-dimensional landslide monitoring system based on an integrated “space–air–ground–underground” approach (Figure 3). By consolidating diverse monitoring technologies—including unmanned aerial vehicles equipped with high-speed cameras, digital cameras, and 3D laser scanners—the system achieved comprehensive, high-precision, and real-time monitoring of the Xinpu Landslide in the Three Gorges area. The results indicate that shallow soil temperature is closely correlated with external climate conditions, exhibiting significant variations in response to seasonal cycles. This finding can provide critical technical support and valuable references for landslide hazard prevention and mitigation. In Dongguan City, Zhong [31] established professional monitoring stations at hidden hazard points characterized by high risk and severe potential consequences. The monitoring system primarily employs a real-time video network to implement a station-network monitoring approach. This system enables automated monitoring and real-time data transmission, capable of providing early warnings prior to disaster occurrence, thereby effectively reducing the risks of casualties and economic losses. Huang [32] implemented an integrated monitoring system for the Lishan Landslide in Xi’an by combining video surveillance equipment (e.g., all-weather night-vision cameras, monitoring poles) with other instruments such as rain gauges and crack meters. This system facilitates remote real-time monitoring, thereby supplying long-term, continuous, and reliable data for research on the landslide’s deformation and failure mechanisms. Zhao [33] established a monitoring system incorporating video acquisition devices to conduct real-time monitoring of the Wangmo River debris flow from three perspectives: hydrological conditions, kinematic characteristics, and solid material composition. Analysis of the monitoring data and change trend graphs accurately reflects the dynamic changes within the Wangmo River watershed. Taking the Duwen Expressway as a case study, Lv [34] developed an intelligent video monitoring system for geohazards. The system successfully detected the occurrence of geological events and demonstrated significant effectiveness in its application. In summary, real-time video monitoring technology has demonstrated significant potential for application in geohazard monitoring and early warning. It not only enhances monitoring efficiency and accuracy but also provides critical technical support for disaster prevention and mitigation strategies.

#### 2.2.3. UAV Remote Sensing Monitoring

The rapid advancement of Unmanned Aerial Vehicle (UAV) technology has led to its unique value in the field of geohazards [35]. UAV-based remote sensing is a non-contact method for acquiring surface information, which utilizes UAVs equipped with sensor payloads such as digital cameras, high-definition video cameras, and laser scanners [36]. UAV remote sensing offers rapid data acquisition and extensive spatial coverage. When integrated with data processing and analysis techniques, it proves highly capable of fulfilling geohazard monitoring tasks [37,38]. Moreover, its strong mobility and low-altitude flight capability allow it to overcome limitations inherent in aerial photography and satellite remote sensing, such as frequent cloud obstruction. These advantages establish UAV remote sensing as a crucial component of the remote sensing arsenal [39,40]. Taking Qingchuan County, Guangyuan City as a case study, Wang [41] utilized an unmanned aerial vehicle (UAV) equipped with a non-metric camera to acquire video data of the disaster-affected area. This data was used to construct a 3D model and conduct a landslide susceptibility assessment. The results demonstrate that UAV-based oblique photogrammetry data can serve as an effective method for landslide susceptibility evaluation. Meng [42] conducted photographic observation of a landslide in Yiling District, Yichang City, Hubei Province, utilizing an Unmanned Aerial Vehicle (UAV) equipped with a high-precision gimbal-mounted camera. The acquired data exhibited high reliability, and this method significantly enhanced both the operational efficiency and monitoring accuracy of the landslide investigation. Zhang [43] employed UAV aerial surveying to monitor the Dongzimen landslide. Their study demonstrated the feasibility of using UAV-based oblique photogrammetry for monitoring slope deformation. Wang [44] conducted UAV photogrammetric surveys over the Laohuzui landslide area. By integrating image data processing techniques, they were able to rapidly reconstruct a 3D model of the surveyed area, providing a 360-degree panoramic representation of the actual scene surrounding the geohazard. Vivaldi, V. [45] integrated UAV-based RGB photogrammetry and infrared thermography to investigate a landslide in the Northern Apennines. This combined approach facilitated effective monitoring of the landslide’s activity state and spatial distribution. Taking the Qinglinggou slope in the Three Gorges Reservoir Area as a case study, Huang [46] established an integrated UAV remote sensing monitoring system capable of image acquisition, processing, and identification. This system provides a valuable reference for monitoring steep slopes. Lian [47] employed UAV-based aerial photography in the Taohuagou area. Using a visual interpretation method that integrates primary and auxiliary data, they achieved rapid and accurate identification of landslides, collapses, and cracks. Based on UAV oblique photogrammetry integrated with field investigations and DEM data, Ma [48] interpreted geohazards in a mining area of Liaoning Province and completed a risk assessment of geohazards for the study area. The aforementioned studies demonstrate that UAV remote sensing technology exhibits significant application value in geohazard monitoring and assessment. This technology not only provides robust technical support for rapid response and accurate evaluation of geohazards but also supplies a critical scientific basis for disaster prevention and emergency management.

## 3. Video Image Processing Techniques

### 3.1. Machine Learning-Based Video Image Processing Techniques

Machine learning leverages algorithmic processes to automatically extract patterns from datasets, enabling predictive modeling and decision-making for unseen data in geohazard applications [49]. Deep learning, a key subfield of machine learning, focuses on constructing multi-layer neural networks that autonomously learn hierarchical feature representations from large-scale datasets. Through successive layers, lower-level features are progressively extracted and combined to form more abstract, high-level representations, facilitating the modeling and analysis of complex data structures [50]. Advances in machine learning have led to its widespread adoption in video and image processing, significantly improving the efficiency and accuracy of geohazard monitoring and early warning systems. This subsection provides a comprehensive review of recent applications of machine learning–based video and image processing techniques in this domain.

#### 3.1.1. Convolutional Neural Networks

Convolutional Neural Networks (CNNs), a class of feedforward neural networks tailored for image processing and computer vision tasks, excel at capturing local image features through convolutional operations. They achieve feature dimensionality reduction and noise robustness via pooling layers, and perform deep integration and analysis of features through fully connected layers. This hierarchical architecture enables highly efficient extraction and utilization of image information [51,52].

The application of Convolutional Neural Networks (CNNs) facilitates the accurate delineation and quantification of key indicators, including surface cracks, in landslide monitoring. This approach offers a robust technical foundation for enhancing landslide early warning capabilities. Leveraging the Mask R-CNN model alongside image processing, Vinh Nguyen [53] developed a real-time video-based landslide early warning system (LEWS) to monitor retaining wall damage by identifying and calculating crack widths. The system’s efficacy is high, as evidenced by a displacement measurement accuracy of 84.0–99.4%, a model mAP of 0.86–0.90, an F1-score of 0.83–0.85 for crack identification, and a crack size determination accuracy of 85.0–98.8%. This technique significantly advances the accuracy of landslide monitoring by providing precise data, thereby boosting both automation and early warning precision.

In debris flow monitoring, the application of Convolutional Neural Networks (CNNs) has introduced novel breakthroughs for early detection and early warning. Wu [54] developed a CNN-based system for the detection and identification of debris flow hazards. The system was evaluated using data from seven debris-flow-prone regions in China, including the Peilonggou and Tianmo Gully in Tibet. On the test dataset, it achieved an AUC of 86.3% for detection, 83.7% for identification, and an overall identification AUC of 88.1%. These results demonstrate the system’s efficacy in reliably detecting and identifying debris flow events from video streams. Leveraging digital image recording devices, Pham, M.-V. [55] developed a CNN model based on the YOLO framework for detecting and localizing debris flows. The proposed model demonstrates high accuracy and rapid processing speed, making it well-suited for real-time monitoring and early warning systems, thereby providing a reliable basis for debris flow hazard management. The above outcomes underscore that CNNs significantly improve the precision of debris flow monitoring and provide a critical foundation for disaster early warning.

Additionally, Patil, M.D. [56] synthesized various methodologies for disaster detection, retrieval, summarization, and analysis using information retrieved from diverse platforms such as social media and satellite imagery. Among these, Convolutional Neural Networks (CNNs) form the core of most disaster detection approaches, effectively addressing the limitations of traditional methods. Li [57] established a complete remote monitoring system for highway geological hazards by constructing a framework that synergistically combines Deep CNNs, R-CNN, and manual oversight, using Beijing’s highway network as a case study. This system significantly boosted the identification efficiency and accuracy of hazards, thereby advancing the intelligence of highway maintenance management. Leveraging Convolutional Neural Networks (CNN) and big data algorithms, He [58] developed a radar-based identification model for geological hazards, which effectively enhanced the identification accuracy. In summary, Convolutional Neural Networks (CNNs) demonstrate significant potential in the processing of video and imagery for geohazards. They have substantially improved the accuracy and efficiency of hazard monitoring, providing robust support for enhancing the intelligence level of disaster prevention and control systems.

#### 3.1.2. Other Machine Learning Architectures

Beyond Convolutional Neural Networks (CNNs), a diverse array of machine learning methodologies has been extensively utilized in video- and image-based monitoring and early warning systems for geological hazards. These techniques have demonstrated their respective merits across a variety of application scenarios. Hu [59] proposed a debris flow scene recognition method leveraging the Temporal Segment Networks (TSN) framework with a ResNet-50 backbone. The study demonstrated a significant enhancement in the identification accuracy of debris flow dynamics in video footage from specific cameras under defined conditions, offering a valuable reference for camera-based debris flow monitoring and early warning initiatives. Wang [60] conducted a study in Luding County, Sichuan Province, China, where an integrated approach combining YOLO and U-Net was developed to extract multi-scale features from landslide imagery. This method significantly enhanced landslide identification capabilities and proved effective in mitigating both false positives and omissions in landslide detection. Zhou [61] developed YOLOv8m-GCSlide, an enhanced model based on the YOLOv8 framework. When tested in complex terrain, it demonstrated high performance (e.g., 82.3% recall, 4.2% false alarm rate, 240.6 FPS), indicating its global attention mechanism and loss function effectively capture debris flow features. The incorporated model compression technique successfully balanced accuracy with efficiency, making it a highly promising solution for low-latency, end-device deployment in geologic hazard early warning systems. Li [62] leveraged a combination of a Transformer architecture and the DeepLab v3 model to perform feature extraction and matching for hazard monitoring of the dangerous rock zone in Qutang Gorge’s ancient plank road, achieving comprehensive, targetless displacement monitoring. Chandra, N. [63] conducted a quantitative assessment of satellite and UAV imagery from the Bijie landslide (China) and a landslide in Nepal using four variants of the YOLOv5 model integrated with different attention mechanisms. The results demonstrated that the attention-enhanced YOLO models can accurately update landslide inventories, thereby providing a reliable basis for landslide susceptibility zoning and hazard mapping. Zhang [64] utilized the U-net architecture for image segmentation and feature extraction, achieving a geological hazard detection accuracy of 85.44% and a classification accuracy of 76.13% for debris flows versus landslides, offering a new approach for hazard management. Collectively, these studies highlight the diverse and powerful capabilities of machine learning architectures in the analysis of video and imagery for geological hazards. They have significantly expanded the technical toolkit for monitoring and early warning, thereby contributing novel solutions and insights to disaster risk management strategies.

Based on previous research, this article summarizes the advantages and limitations of the CNN model and other machine learning models applied to video and image processing, as presented in Table 1. Compared with conventional methods, machine learning provides a diverse set of technical approaches for video and image analysis. By selecting models appropriate to specific task characteristics, these techniques can substantially improve processing accuracy and efficiency. In the context of geological hazard monitoring and early warning, machine learning-based video and image processing offers superior monitoring precision, earlier warning capabilities, reduced costs, and continuous 24/7 automated operation, thereby enhancing disaster prevention and mitigation technologies and driving advancements in this field.

### 3.2. Multi-Source Data-Fused Video Image Processing Techniques

Multi-source data fusion refers to the process of integrating information from diverse sources using mathematical models and technical tools to produce high-quality, actionable information. Compared with single-source data processing, it offers advantages such as enhanced detectability and reliability, expanded spatiotemporal coverage, improved detection accuracy, richer feature dimensionality, and higher spatial resolution [65]. Multi-source data fusion can be implemented at the data level or through the fusion of data processing techniques. When applied to geohazard monitoring and early warning, it enables more accurate and reliable operational outcomes. This subsection synthesizes global research on methodologies for geohazard monitoring and early warning based on multi-source data fusion.

#### 3.2.1. Data-Level Fusion

**Figure 4:**
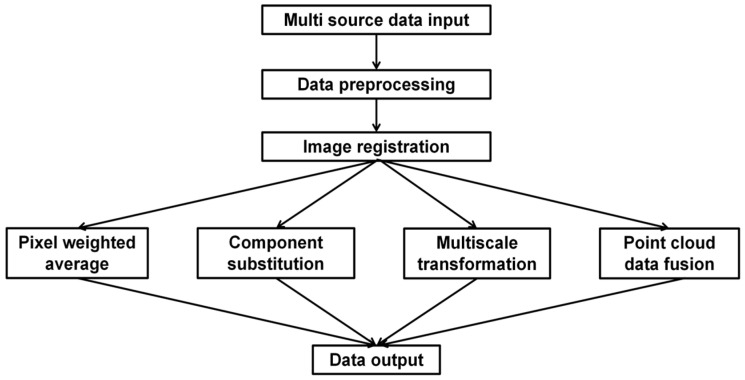
Data layer integration flow chart.

Data-level fusion represents the most fundamental tier of multi-source data integration. Its core principle is the direct combination and processing of raw, unprocessed data from heterogeneous sources, thereby preserving the finest details and achieving a “consistent integration” of the original datasets. As illustrated in Figure 4, data-level fusion involves registering multi-source raw data and integrating it using techniques such as pixel-weighted averaging and multi-scale transformation, resulting in a new dataset with higher information density and greater consistency.

In geological hazard monitoring and early warning, multi-source data fusion has emerged as a crucial approach for enhancing precision and efficiency. By integrating diverse data sources—including satellite imagery, UAV data, video footage, and ground sensor readings—this method provides a comprehensive understanding of hazard dynamics, thereby supporting timely alerts and accurate risk assessments. Deng [66] integrated multiple data sources, including remote sensing imagery, unmanned aerial vehicle (UAV) photographs, and video footage, from the Xidongpu catchment on the left bank of the Yarlung Zangbo River in the southeastern Tibetan Plateau. This comprehensive dataset documented the entire process of a debris flow, thereby providing critical evidence for analyzing the dynamic characteristics of such hazards. Yang [67] improved the GSI assessment by fusing VIS-NIR hyperspectral imagery and digital panoramic borehole data. By calculating strength reduction and integrity coefficients from carbonate content and fracture area ratio, the method achieved a high monitoring accuracy for rock mass degradation, with a GSI error rate below 10%. Following the Wenchuan earthquake, Zeng [68] developed an approach for rapid disaster assessment by integrating multi-source remote sensing data (e.g., satellite, aerial, low-altitude imagery) to produce high-quality DOMs and DEMs. The automated extraction of large-scale collapse masses and generation of interpretation maps enabled efficient and accurate damage information acquisition, which served as a critical scientific basis for rescue operations and reconstruction planning. In response to the frequent slope collapses caused by unstable rock zones in Guizhou, Zhang [69] enhanced the conventional Incremental Vector Method (IVM) by integrating GNSS, sensor, video, aerial, and satellite data, and incorporating two key quantitative indicators: displacement angle and fracture width. This approach elucidated the rock mass’s mechanical properties, identifying precursors for similar geo-hazards in the region. Aiming at the potential geohazards triggered by rock mass degradation (RMD) within the water-level fluctuation zone of the Three Gorges Reservoir area, Dai [70] integrated orthophotos, multi-angle 3D oblique photography, and high-precision Digital Elevation Models (DEMs). Through image processing and data analysis, 116 new potential RMD-induced geohazard sites were identified, thereby achieving the early identification of latent geological risks. Wang [71] developed a real-time monitoring system based on video imagery, integrated with other remote sensing data, to achieve dynamic monitoring and early warning of geological hazards. Zhao [72] established a multi-source data-driven geological hazard monitoring and early warning system for Deqin County, Yunnan, by integrating 3D laser scanning, InSAR, GNSS, UAV and IoT data. The system features information query and analysis, risk assessment and simulation, and early warning capabilities, thereby improving the intelligence, informatization, and dynamic monitoring of geohazards in alpine gorge areas. For the early identification of significant geological hazard potentials, Xu [73] developed an integrated space–air–ground observation system by combining data from spaceborne (high-resolution optical and InSAR), airborne (e.g., LiDAR, UAV photogrammetry), and terrestrial (e.g., surface, internal slope monitoring) platforms. In summary, multi-source data fusion diversifies methodological approaches for geohazard monitoring and substantially improves both accuracy and efficiency, providing a critical foundation for precise early warning systems and science-based risk management.

#### 3.2.2. Fusion of Data Processing Techniques

**Figure 5:**
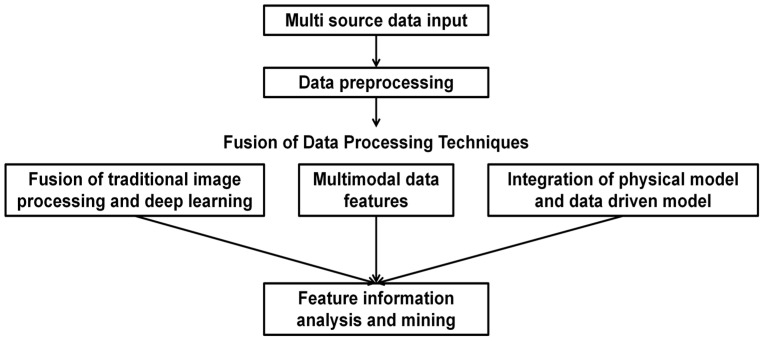
Data processing technology integration process.

As illustrated in Figure 5, data processing technology fusion accurately extracts subtle and fuzzy features from raw data by integrating multiple technical approaches. Through collaborative processing, it effectively addresses the heterogeneity, uncertainty, and complex correlations inherent in multisource data, thereby significantly enhancing the accuracy and reliability of data fusion.

**Figure 6:**
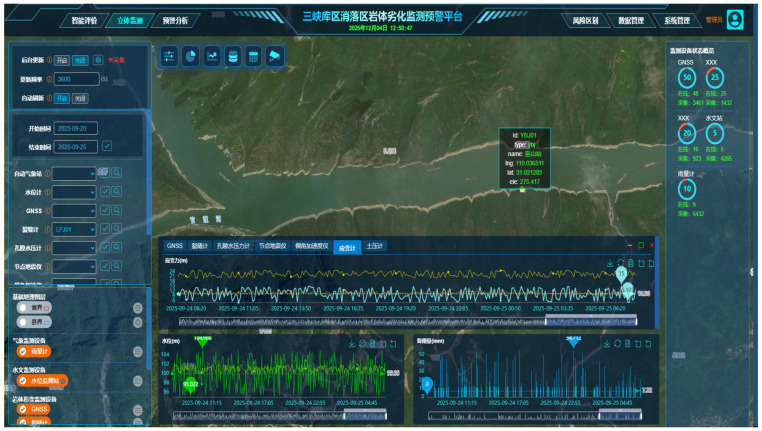
Geological Hazard Monitoring and Early Warning System for Three Gorges Reservoir (The interface displays a satellite-image-based geographic environment combined with real-time monitoring data. Multiple panels provide visualizations of displacement time series, vibration/acceleration signals, rainfall and hydrological indicators, as well as sensor status information. The system supports multi-source data integration, spatial positioning of monitoring points, and dynamic trend analysis to assist in landslide stability assessment and early-warning decision-making).

In geological hazard monitoring, the fusion of multi-source data processing techniques has emerged as a crucial approach for improving monitoring precision and enhancing early warning capabilities. Liu [74] utilized multi-source data, including satellite imagery, optical images, and UAV field surveys, in conjunction with the Mass Point Method (MPM) and Cross-Correlation Function (CCF), to investigate the reactivation mechanism of a landslide. Shi [75] designed an intelligent monitoring system by integrating GNSS displacement monitoring technology, IoT sensing technology and video analytics technology, which effectively addressed the requirements for slope monitoring. For research on slope monitoring systems, Deng [76] developed a video image-based geological hazard monitoring and early warning system. Utilizing acquired video data, the system employs background subtraction, median filtering, mathematical morphology, and Kalman filtering for data processing. It enables multi-index monitoring of initiation points for various hazards, such as landslides and debris flows, facilitating the automatic issuance of more accurate alerts and providing a foundation for intelligent data analysis. Chang [77] employed DFT and DST to generate spectrograms for landslide feature identification, utilized low-frequency signals to invert the direction and magnitude of the forcing mechanisms, and combined cross-correlation methods with amplitude attenuation analysis to locate the landslide source. This methodology provides a basis for improved landslide monitoring and risk management. By integrating Measurement-While-Drilling (MWD) data and Principal Component Analysis (PCA), and calibrating the results with borehole video, UAV 3D reconstruction, and cuttings assays, Juan Navarro [78] established a classification model for rock mass degradation. This model facilitates the automated assessment of rock mass structure, strength, and the identification of mining waste. For the Bijiashan rockfall event in the Three Gorges Reservoir area, China, Wang [79] utilized UAV-based 3D modeling to delineate zones of rock mass degradation. Video target tracking technology was employed to dynamically monitor the displacement, velocity, and acceleration of marked points. By integrating coordinate correction and non-linear scale transformation, parameters characterizing the degradation process were extracted, enabling the quantification of the chain evolution of the collapse and its associated energy release. In order to respond the challenge of “wide range, complex causes and difficult monitoring” of geological disasters in the reservoir area, Tang [80] integrated multiple technologies, including InSAR, GNSS, deep-seated displacement monitoring, groundwater level and flow rate measurements, and video surveillance, to construct an intelligent monitoring and early warning system for reservoir-related geological hazards. In this system, different monitoring technologies are given clear roles according to their characteristics, and together form a three-dimensional perception network, which realizes the remote all-weather monitoring and early warning of the integration of sky and earth. Figure 6 shows an intelligent monitoring system that integrates functions such as intelligent evaluation, three-dimensional monitoring, warning analysis, and risk zoning. Overall, the fusion of diverse data processing methods enables comprehensive analysis of hazard characteristics, providing a scientific foundation for risk mitigation and emergency response, and advancing the field of geohazard monitoring and early warning.

## 4. Limitations and Future Research Directions

### 4.1. Limitations of the Video-Based Monitoring System

Driven by rapid technological advancements, video-based monitoring systems have been widely adopted in geohazard monitoring and early warning. However, despite their notable advantages in real-time observation and visualization, several limitations remain in practical applications. These shortcomings constrain the overall effectiveness and reliability of video-based approaches in geohazard monitoring and early warning. This section discusses these limitations in detail, with particular emphasis on data storage and transmission, environmental adaptability, and monitoring coverage. The objective is to provide a reference and source of insight for related research and practical implementation.

1. Data Storage and Transmission: Video-based monitoring systems generate massive volumes of data, placing significant demands on data transmission [81]. For example, 24-h continuous monitoring of multiple potential landslides in a mountainous area can produce petabyte-scale datasets, potentially causing transmission delays that compromise real-time performance. In addition, the substantial storage requirements lead to high operational costs. To address these challenges, a “cloud–edge–end” collaborative computing framework can be implemented, performing data preprocessing and computational analysis at both the device and monitoring site levels, thereby alleviating transmission bottlenecks and reducing computational load.
2. Environmental Adaptability: Geohazard-prone areas are often subject to harsh weather conditions. Heavy rain, dense fog, intense snowfall, and dust storms can obscure camera views, resulting in image blurring, reduced contrast, or complete failure to capture critical details such as landslide cracks or slope deformations [82,83]. Moreover, these hazards frequently occur in complex terrains, including mountainous regions and deep valleys, where line-of-sight obstructions from rocks or cliffs create monitoring blind spots, limiting measurement precision [84,85]. Dense vegetation during summer can further obscure landslide and rockfall masses, preventing optical imagery from capturing deformations in vegetated areas [86,87]. To overcome these limitations and enhance model generalization, multimodal data fusion and cross-modal learning can be employed. Integrating complementary instruments such as infrared thermal imagers and InSAR, combined with deep learning models that correlate visible light, infrared, and radar data, enables cross-modal information enhancement and improves monitoring performance under challenging environmental conditions.
3. Monitoring Coverage: The field of view of video-based monitoring systems is typically limited to the immediate vicinity of the surveillance equipment [88,89], and measurement accuracy is affected by target size and camera acquisition angles [90]. Such systems primarily capture surface phenomena and cannot directly detect subsurface variations, including changes in soil or rock porosity and groundwater levels. They are also often insufficient for detecting subtle precursory deformations in the early stages of geohazard development [91]. To address these limitations, a unified data fusion platform can integrate spatiotemporal surface changes from video analysis, large-scale deformation rates from InSAR, and subsurface parameters from sensors, thereby improving overall monitoring accuracy and coverage.

Moreover, to comprehensively capture potential geohazard sites, the field of view of monitoring systems—such as high-definition cameras and wide-area UAV patrols—inevitably encompasses residential areas, roads, and farmland. As a result, these systems may unintentionally and continuously collect sensitive personal information, raising concerns among the public. To address this issue, privacy-preserving techniques (e.g., face blurring) should be applied during the initial stages of data processing to desensitize identifiable information. Additionally, prior to system deployment, affected communities should be informed about the system’s purpose and the privacy protection measures in place, and deployment should proceed only with the approval of local authorities.

### 4.2. Prospects of the Video-Based Monitoring System

In contemporary society, the risks posed by geohazards to human life and property are becoming increasingly significant. As an essential monitoring tool, video-based monitoring systems have shown considerable potential and promising prospects in geohazard monitoring and early warning. This section provides a comprehensive discussion of the development trajectory of these systems, examining emerging technological trends and future application potential. The aim is to offer valuable references and insights for advancing research and practical implementation in this field.

1. All-weather, omnidirectional, high-precision monitoring. Advances in technology will enable video-based monitoring systems to achieve all-weather, omnidirectional observation, allowing clear monitoring of geological hazards even under adverse environmental conditions [92]. Furthermore, these systems will also be capable of detecting minute changes, such as rock fractures and landslide displacements, providing essential data for quantitative assessment of disaster evolution.
2. Technological integration and intelligent advancement. The integration of Internet of Things (IoT), 5G mobile communications, big data, and cloud computing will enhance video-based monitoring systems’ support for disaster risk reduction decision-making, improving proactive response capabilities [93]. Simultaneously, continuous advancements in artificial intelligence (AI) and machine learning (ML), including deep learning, computer vision, and big data analytics, will strengthen image recognition, real-time analysis, and scene understanding. This technological evolution is expected to enable more efficient and precise applications in geohazard monitoring [94].
3. Space–Air–Ground integrated monitoring. Combining video-based monitoring with satellite remote sensing and sensor networks facilitates the establishment of a Space–Air–Ground integrated early warning system [95,96]. This multi-dimensional, multi-tiered framework provides a more comprehensive and accurate characterization of hazard dynamics, significantly enhancing monitoring and early warning efficiency and precision.
4. Multi-source data fusion. Integrating multi-source data addresses the limitations of individual monitoring techniques, enabling comprehensive, multi-level observation of geological hazards [97,98]. By consolidating and analyzing data from various departments and platforms, this approach helps break down information silos and promotes data sharing, improving both the accuracy and timeliness of early warnings [99]. Ongoing technological advances continue to refine multi-source data fusion methodologies, resulting in increasingly efficient real-time monitoring systems.
5. Integration with emergency communication technologies. Linking video-based monitoring systems with emergency communication networks allows real-time video transmission from disaster sites. This provides visually intuitive information for command and rescue operations, substantially improving the speed and effectiveness of emergency response.

## 5. Conclusions

Rapid technological advancements have highlighted the substantial potential of video-based monitoring systems in geohazard monitoring and early warning. This article reviews their applications from three perspectives: video-based monitoring technologies, machine learning–based video processing techniques, and multi-source data fusion–based video processing techniques:

1. Video-based monitoring technology employs video acquisition devices to capture images of geohazard sites. Owing to its broad spatial coverage, it can visually document the dynamic processes of hazards such as landslides and debris flows, thereby providing essential information for their early identification.
2. Machine learning–based video processing techniques enable the automated analysis of image data, allowing for the identification of critical features such as surface cracks and landslide displacements. These capabilities substantially enhance the efficiency and accuracy of geohazard monitoring.
3. By integrating multi-source data and diverse processing techniques, video-based monitoring systems can operate synergistically with instruments such as GNSS receivers and rain gauges. This integration enables cross-validation of observations, thereby substantially enhancing the reliability of early warning outcomes.

With the support of machine learning methods and multi-source data fusion technologies, video-based monitoring systems can achieve automated and intelligent detection of geohazard features. These advancements greatly enhance monitoring efficiency and compensate for the limitations of single-source video monitoring, particularly regarding measurement accuracy and all-weather operational capability. When integrated with other geospatial datasets, these systems contribute to a more comprehensive, three-dimensional, and reliable early-warning perception network. Nevertheless, current video-image monitoring systems still face several challenges, including limited algorithmic robustness in complex environments, insufficient high-quality training data, constrained model generalization, and inherent trade-offs between real-time performance and accuracy.

Looking ahead, research in this field should continue to advance toward greater algorithmic intelligence, deeper data fusion, and enhanced system-level collaboration. Video-based monitoring systems are expected to become a core component of the “early detection, early warning, and early response” framework for geological hazards. Their integration will accelerate the shift toward intelligent, automated, and grid-based monitoring and early warning architectures, thereby strengthening the protection of human life and property and reinforcing the first line of defense in disaster prevention and mitigation.

## Figures and Tables

**Table 1:** Comparison of different machine learning models.

Methods	Advantages	Limitations
CNN	Strong feature extraction capability and migration capability	Lack of positioning ability and time series modeling ability
TSN	Strong time series modeling ability and suitable for long sequence analysis	Complex calculation and difficult to realize
U-net	Pixel level accurate segmentation and boundary alignment	Absence of temporal modeling capacity and high computational demand
YOLO	High real-time performance and applicable to global reasoning	Rough positioning accuracy and insensitive to small targets

## Data Availability

The data presented in this study are available upon request from the corresponding authors.
